# Antimicrobial Peptides Design by Evolutionary Multiobjective Optimization

**DOI:** 10.1371/journal.pcbi.1003212

**Published:** 2013-09-05

**Authors:** Giuseppe Maccari, Mariagrazia Di Luca, Riccardo Nifosí, Francesco Cardarelli, Giovanni Signore, Claudia Boccardi, Angelo Bifone

**Affiliations:** 1 Center for Nanotechnology Innovation @NEST, Istituto Italiano di Tecnologia, Pisa, Italy; 2 NEST, Scuola Normale Superiore and Istituto Nanoscienze-CNR, Pisa, Italy; Max Planck Institute for Biophysical Chemistry, Germany

## Abstract

Antimicrobial peptides (AMPs) are an abundant and wide class of molecules produced by many tissues and cell types in a variety of mammals, plant and animal species. Linear alpha-helical antimicrobial peptides are among the most widespread membrane-disruptive AMPs in nature, representing a particularly successful structural arrangement in innate defense. Recently, AMPs have received increasing attention as potential therapeutic agents, owing to their broad activity spectrum and their reduced tendency to induce resistance. The introduction of non-natural amino acids will be a key requisite in order to contrast host resistance and increase compound's life. In this work, the possibility to design novel AMP sequences with non-natural amino acids was achieved through a flexible computational approach, based on chemophysical profiles of peptide sequences. Quantitative structure-activity relationship (QSAR) descriptors were employed to code each peptide and train two statistical models in order to account for structural and functional properties of alpha-helical amphipathic AMPs. These models were then used as fitness functions for a multi-objective evolutional algorithm, together with a set of constraints for the design of a series of candidate AMPs. Two ab-initio natural peptides were synthesized and experimentally validated for antimicrobial activity, together with a series of control peptides. Furthermore, a well-known Cecropin-Mellitin alpha helical antimicrobial hybrid (CM18) was optimized by shortening its amino acid sequence while maintaining its activity and a peptide with non-natural amino acids was designed and tested, demonstrating the higher activity achievable with artificial residues.

## Author Summary

In recent years, the increasing and rapid spread of pathogenic microorganisms resistant to conventional antibiotics especially in hospital settings spurred research for the identification of novel molecules endowed with antimicrobial activities and new mechanisms of action. Antimicrobial peptides (AMPs) received an increasing attention as potential therapeutic agents because of their wide spectrum of activity and low rate in inducing bacterial resistance. Currently, research is focused on the design and optimization of novel AMPs to improve their antimicrobial activity, minimize the cytotoxicity and reduce the proteolytic degradation, also in biological fluids. To this end, the introduction of non-natural amino acids will be a key requisite in order to contrast host resistance and increase compound's life. However, the amino acidic alphabet extension to non-natural elements makes a systematic approach to AMPs design unfeasible. A rational in-silico approach can drastically reduce the number of testing compounds and consequently the production costs and the time required for evaluation of activity and toxicity. In this article, AMP in-silico design with non-natural amino acids was performed and a series of candidates were tested in order to demonstrate the potentiality of this approach.

## Introduction

Antimicrobial peptides (AMPs) are small evolutionally conserved molecules found among all classes of life, from multicellular organisms to bacterial cells [1], [2]. In higher organisms, AMPs play a major role in innate immunity as a part of the first defence line against invading pathogens. In bacteria, AMPs provide a competitive advantage for the producer in certain ecological niches as weapons against other bacteria. Alpha-helical AMPs are among the most abundant and widespread membrane-disruptive sequences in nature and represent a particularly successful structural arrangement for innate defense, as it can easily afford peptide insertion into lipid bilayers [3]. In fact, the amphipathic structure facilitates electrostatic interactions between the peptide and the target cell membrane. Completion of the folding process involves hydrophobic interactions between the non-polar residues of the peptide and the hydrophobic core of the lipid bilayer [4], [5]. AMP membrane perturbation activity can be explained by at least three major mechanisms, all leading to bacterial membrane's collapse and subsequent cell's death. Two of these models (i.e. the ‘barrel-stave’ and the ‘toroidal-pore’ models) rely on the peptide ability to form ordered transmembrane channels/pores, while the so called ‘carpet model’ implies that, at a critical threshold concentration, the peptides disrupt the bilayer in a detergent-like manner, eventually leading to the formation of micelles [6]. In recent years, AMPs are actively researched not only as direct antimicrobial agents, but also as potential endosomolytic moieties promoting the release of biomolecules into cells for delivery purposes [7]–[9]. On the other hand, the increasing prevalence of antibiotic resistance necessitates the development of new ways to combat bacterial infection. Although some AMPs are already in clinical and commercial use (see Table S1 for a list of AMPs commercially available and in clinical trial), the future design of novel AMPs will need to minimize the toxicity against eukaryotic cells and enhance the resistance to proteolytic degradation, with a key opportunity being offered by the introduction of non-natural amino acids (AA) to contrast host resistance and increase compound's life.

Thus far, several methods have been proposed for the rational design of AMP sequences with improved activity. In particular, computer-aided identification and design of AMPs played a crucial role in this area [10]. Most of these approaches are based on sequence alignment and calculation of amino acidic frequencies (e.g. template-based approaches and some machine-learning methods) [11]–[14]. However, several major disadvantages may limit the potential of these strategies. For instance, there is no understanding of the physicochemical requirements that play a crucial role in regulating the peptide activity. Also, peptide design is limited by the use of natural AAs only. In the effort to overcome these limitations, quantitative structure-activity relationship (QSAR) descriptors have been employed to correlate primary AA sequence with a given biological activity [15], [16]. QSAR descriptors provide a reliable statistical model for prediction of the activities of new chemical entities. In particular, *Hellberg et al.* developed the so called ‘z-scale’ descriptors [17], highly condensed variables derived from a principal component analysis (PCA) of several experimental or theoretical physicochemical properties for the 20 naturally occurring AAs. In detail, these z-scale descriptors correspond to the first three principal components explaining the variance in the set: z_1_, z_2_, and z_3_ represent the AA hydrophobicity, steric properties, and polarity, respectively. QSAR analysis of peptides using these descriptors has proven effective in predicting different physiological activities [18]–[20]. Z-scale descriptors were also successively expanded to include artificial AAs [21]. The new z-scales include 87 AAs and two extra variables (z_4_, z_5_) describing the electronic effects of the residues.

All the mentioned descriptors combined with statistical analysis allow the design of novel AMP and the optimization of already existing ones in terms of desired characteristics such as improved activity, decreased toxicity, and easy synthesis. Due to the huge number of possible amino-acids combinations, stochastic optimization methods such as Genetic algorithms (GA) may be a preferred tool to perform directed random searches in large problem spaces, such as those encountered in drug design [22], [23]. In particular, when simultaneous optimization of two or more characteristics is required, a class of GA called multi-objective evolutional algorithms (MOEA) can be used to provide an optimal solution [24] [25].

In order to overcome current limitations and develop a flexible computational approach for AMP design, able to account for non-natural AAs, here we combine the representation of antimicrobial peptides in terms of physicochemical features with genetic algorithms. The novelty of this approach is in the unified treatment of both natural and non-natural AAs, allowing a systematic exploration of this enhanced combinatorial space. We target our approach to alpha-helical AMPs because of their advantages in terms of biological activity, mechanism of action, and ease of synthesis and manipulation [3]. Rather than training a single model on the existing alpha-helix AMPs we separately addressed the structure and the function characteristics, combining the two aspects in the design phase. Thereby, starting from two different sets of existing peptides, two statistical models were trained in order to account separately for structural and functional characteristics of alpha-helical AMPs. This approach has the advantage of considering broader and unbiased datasets, with respect to an alpha-helix only AMP dataset, enhancing the performance of the training phase. The first model represents antimicrobial physicochemical properties and was trained on a set of AMPs taken from the literature. The second model accounts for the all-helix conformation of the peptide and is based on a non-redundant set of all-alpha helix protein fragments. This *in silico* approach was used to design a set of five peptides with natural AAs (GMG_01, GMG_02, GMG_03, GMG_01_SCR, CM_12_). GMG_01 and GMG_02 were designed ex-novo and predicted to be antimicrobic, while GMG_03 and GMG_01_SCR were designed as negative controls with no predicted bactericidal activity. CM_12_ was obtained by the reduction and optimization of the CM_18_ (Cecropin (1–7)-Melittin (2–12)) sequence, a well-characterized antimicrobial peptide. Finally, an AMP sequence containing non-natural AAs (GMG_05Z) was designed. Antimicrobial properties of all these peptides were experimentally validated *in vitro* by testing the minimum bactericidal concentration (MBC) against *S. aureus* and *P. aeruginosa* strains, representative of Gram-positive and Gram-negative bacteria respectively. In addition, Molecular Dynamic (MD) simulations were performed to predict and analyze the structural properties of the designed peptides, in particular to confirm the presence of helical motives.

## Results/Discussion

### Peptide encoding

Two different sets of peptides were prepared in order to represent functional and structural characteristics of alpha-helical AMPs. Dataset A, representing the functional requirements for AMP activity, was accurately compiled from the literature of existing and characterized antimicrobials. Dataset B accounts for the structural characteristics of alpha-helix AMPs and was assembled from a well-defined non-redundant set of proteins. Two types of descriptor encoding were utilized in order to present the training datasets to the learning algorithm (see **Table 1**): global descriptors and topological descriptors. Global descriptors are variables representing the whole molecule, while topological descriptors are variables representing the interaction of different residues along the amino acidic sequence. Charge and hydrophobicity related characteristics are among the most important properties for active peptides. [26], [27]. Indeed, positively-charged peptides, rich in basic residues, particularly Lysines, can insert into the bacterial membrane more easily [5], [28]. Hydrophobicity determines folding, binding to receptors, and interactions of proteins and peptides with biological membranes. Z-scale averages moments (**Equation 2** in Materials and Methods) are used to account for hydrophobicity, as well as polarity and steric effects of each peptide.

**Table 1:** List of descriptors.

Type	Abbreviation	Description	N
Global	NetCharge@5	Net charge at pH = 5.	1
	NetCharge@7	Net charge at pH = 7.	1
	NetCharge@9	Net charge at pH = 9.	1
	pI	Isoelectric point.	1
	AA Count	Total amino acid count.	1
	chPos	Sum and average of positive charges in side chain.	2
	chNeg	Sum and average of negative charges in side chain.	2
	Z_i_ Average	Z-scale average sum along peptide sequence.	5
	μZ_i_	Z-scale moment distribution along peptide sequence.	5
Topological	AC	Min and Max auto covariance values between the same descriptor.	100
	CC	Min and Max cross covariance values between two descriptors.	400

Two classes of descriptor were used in order to describe a single amino acidic sequence: global descriptors and topological descriptors. Here are listed for each class the type and number of variables.

Topological description of the peptide sequence was accounted for by encoding QSAR descriptors into auto- and cross covariance (ACC) values. Classical ACC transformation was introduced by Wold *et al* [29] and results in two kinds of variables: auto covariance (AC) of the same descriptor and cross covariance (CC) between two different descriptors. Briefly, for a given protein sequence, ACC variables describe the average interactions between residues distributed a certain *lag* apart throughout the whole sequence. Besides describing the sequence order, ACC has the ability to transform each AA sequence of variable length into uniform equal-length vectors. This feature is very important in data mining methods, where a fixed-length vector describing each instance is required. However, averaging along the entire sequence may cause loss of information about strong and weak correlations. To cope with these limitations, the *Maximum of auto- and cross-covariances* (MACC) algorithm was introduced [30], where positive and negative descriptor values are considered separately and only the maximum value of each lag is used. In this work we introduce an ACC descriptor accounting for both weak and strong correlations, the Minimum and Maximum of auto and cross-covariances (mMACC) descriptor (**Equation 1**). 
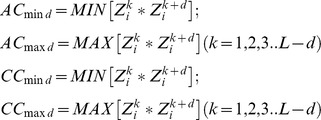

**Equation 1**. Minimum and Maximum of auto and cross-covariance equations.

Where *Z^k^_i_* is the *i*-th descriptor of residue *k* in the sequence, *d* is the lag. As in the MACC algorithm, the maximum value of each interaction is taken into account. However, in the mMACC each z-scale descriptor is shifted by the absolute minimal value in order to have only positive interactions. This reduces the number of combinations, while maintaining both information of strong and weak interactions. In the following, the newly introduced ACC descriptor performances are compared with classical ACC and MACC descriptors.

### Feature selection and model training

**Figure 1:**
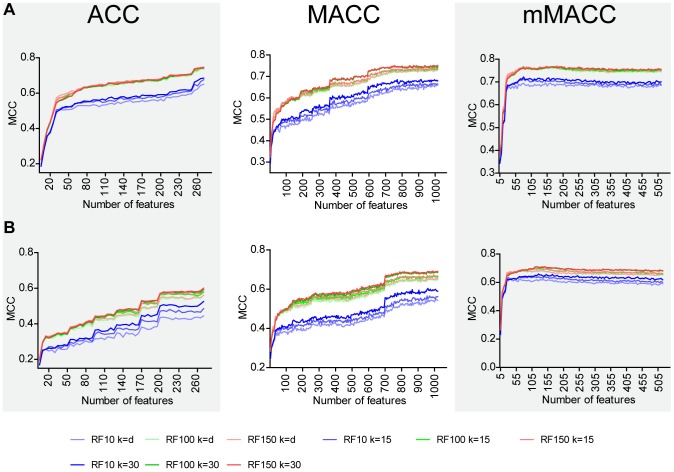
IFS plot. Graph showing the change of the MCC values versus the feature numbers in each trained model for each encoding. For RF training, nine different combinations of variable were tested: M = 10, 100 or 150 is the number of trees in the RF and T = d, 15 or 30 is the number of features assigned to each tree (d is the default value of logM+1). In each model, the mMACC algorithm performed better both in the absolute MCC value, and in the (smaller) number of features. A) In the AMP model, the MCC value reached the peak with M = 150, T = 30 when the number of features = 200. B) In the all-alpha model, the MCC value reached the peak with M = 150, T = 30 when the number of features = 215. For both models, the features thus obtained were used to form the optimal feature set for the training phase.

In the selection of the descriptors a tradeoff should be found between the performance of the encoding (i.e. how well the statistical model based on a particular encoding is able to predict the peptide alpha-helix structure and/or antimicrobial activity) and the requirement of minimizing the number of descriptors. Indeed, on equal terms of performance, a lower number of features is preferable, since the resulting model is less computationally expensive and the interpretation of resulting models is simpler. **Figure 1** reports the performance of the three encodings (ACC, MACC, mMACC) as a function of the number of descriptors used. Prior to this analysis the descriptors were ordered by the mRMR (minimum redundancy maximum relevance) algorithm [31]. The performance is evaluated by the *Mathews correlation coefficient* (MCC) (**Equation 4** in the Materials and Methods), which assesses the prediction in terms of true and false positives and negatives. The maximum MCC value, corresponding to the optimal feature set, was compared for each encoding, as shown in **Table 2**. The mMACC algorithm performed better both in the absolute MCC value, and in the (smaller) number of features. On the basis of these preliminary tests, the mMACC algorithm was chosen to encode topological descriptors for both Dataset A and Dataset B. mMACC plot of Dataset A showed a peak at 200 descriptors, whereas Dataset B reached its maximum of accuracy at 215, and these subsets were selected to construct each training model. Results are summarized in **Table 3** and the complete lists of features are reported in Tables S2 and S3. Hereafter, the genetic algorithms for peptide sequence prediction will be based on these final optimal features. The distribution of the selected descriptors in terms of z-scales interactions and as a function of the lag between AAs is reported in the SI (**Figure S4, Text S1**).

**Table 2:** ACC descriptors performance.

Descriptor	Max Features Number (topological+global)	Dataset A		Dataset B	
		MCC	# Features	MCC	# Features
ACC	250+19	0.75 (std: 0.04)	270	0.60 (std: 0.04)	269
MACC	1000+19	0.75 (std: 0.05)	1020	0.69 (std: 0.05)	1019
mMACC	500+19	0.77 (std: 0.04)	200	0.69 (std: 0.04)	215

The maximum MCC value, corresponding to the optimal feature set, was compared for each encoding.

**Table 3:** Final datasets statistics.

Dataset	Accuracy	Sensitivity	Precision	MCC
Dataset A	0.898	0.776	0.912	0.77 (std: 0.04)
Dataset B	0.871	0.726	0.84	0.69 (std: 0.04)

Each dataset was trained with its optimal features set and performance was measured. Dataset A represents AMPs, while Dataset B represents all-alpha helical peptides.

**Figure 2:**
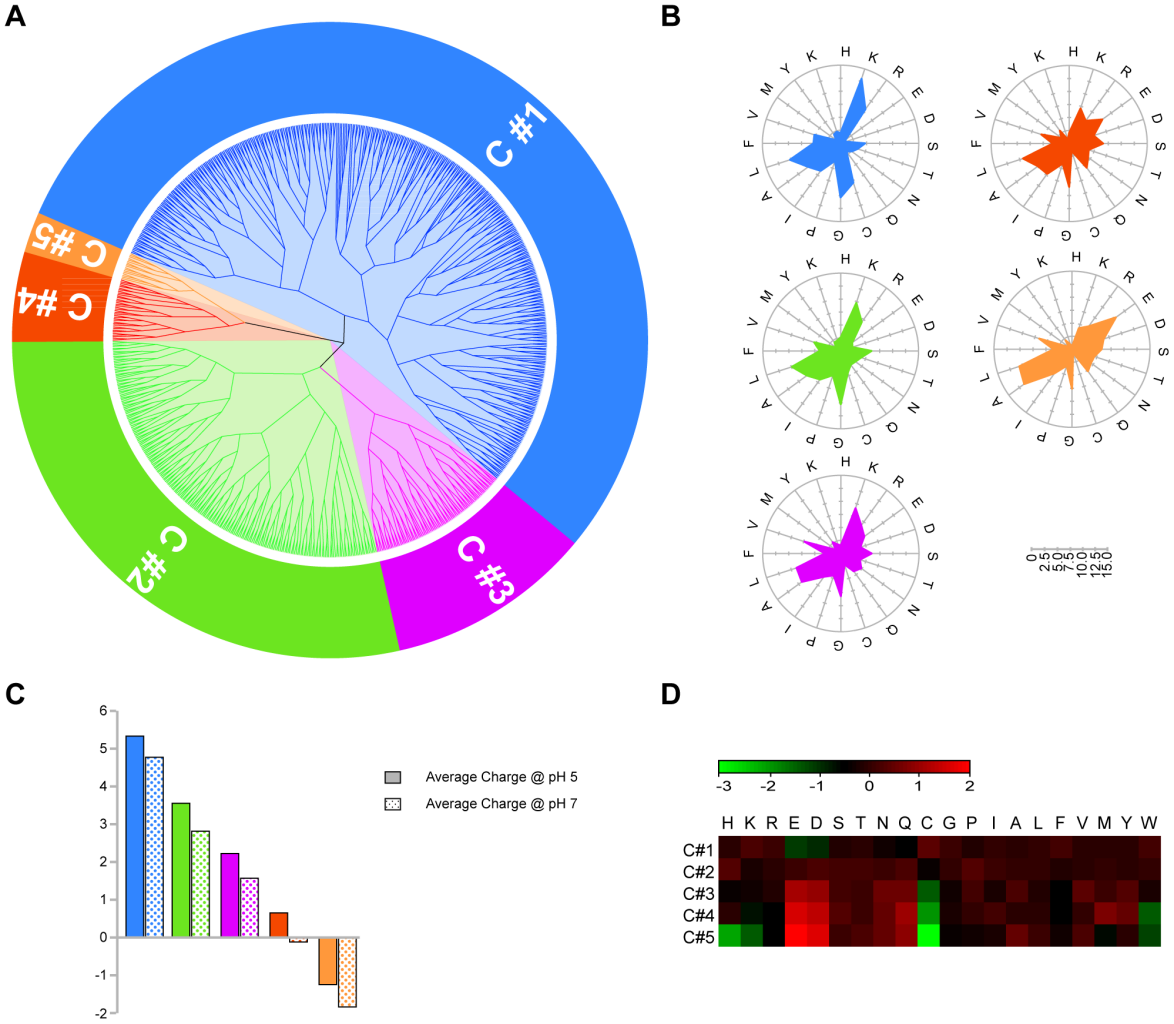
RF proximity cluster analysis. A) Dendrogram representing the cluster subdivision of AMPs dataset, based on RF proximity values. B) Radar distribution of cluster AA composition (i.e. count of each AA normalized by the total number of AA in the cluster). C) Average net peptide charge of each cluster at different pH conditions. D) Heat map representing relative abundance of each AA, calculated as Log_2_ of AA composition in the cluster normalized by AA composition of the entire AMP set.

RF has several properties that allow extracting relevant trends from data with complex variable relations. Proximity values are a measure of similarity between samples, calculated as the number of times the two samples end up in the same terminal node of the tree [32]. In this way, subclasses can be identified by finding peptides that have similar proximities to other AMPs. A matrix representing proximity values of each AMP in Dataset A was obtained from the final model. Cluster analysis resulted in five different clusters and the distribution of relevant properties was analyzed, as shown in **Figure 2**. A dendrogram represents the subdivision into clusters (**Figure 2A**), while a radial distribution of AA frequency is shown in Figure 2B, reflecting the average net charge at different pH (**Figure 2C**). A heatmap showing the AA relative abundance of each cluster is represented in panel D. Cluster1 and Cluster2 present a high average net charge, with a different distribution of the charged residues. In particular, Lysine appears to be more frequent in Cluster 1, as shown in the heatmap. Clusters 3, 4 and 5 present a lower net charge, due to the higher abundance of negatively charged residues. This analysis stresses the role of overall charge and AA composition in classifying various AMP families.

### Peptide ab-initio design

The algorithm can be asked to select sequences optimizing a particular property, in this case, presence or absence of antimicrobial activity and secondary structure. Two *ab initio* AMPs were chosen from two different trial sessions, hereafter named GMG_01 and GMG_02. As a control, a session was performed aiming to the selection of non-antimicrobial, alpha-helical peptides (GMG_03). A second control was synthesized, GMG_01_SCR, a scrambled version of GMG_01 obtained by selection of the sequence with the lowest fitness upon permutation of the original sequence. This control was chosen in order to assess whether the algorithm was able to account for sequence order. After synthesis and purification of the above-mentioned peptides, MBC tests were performed in triplicate on *S.aureus* and *P.aeruginosa* ATCC strains. Peptides sequences, prediction scores and MBC values are summarized in **Table 4**. GMG_01 and GMG_02 yielded an antimicrobial activity comparable to the most effective antimicrobial peptides described in literature. As expected, GMG_01_SCR yielded an antimicrobial activity approximately 16 times lower than the parental one, demonstrating that the AMP prediction model accounts for peptide's AA sequence. The residual antimicrobial activity was probably due to the reduced size of the peptide and the overall cationic nature of GMG_01_SCR. In fact, a short sequence with repeated AAs is not likely to present significant differences in its primary structure; consequently, its chemophysical profile results similar to the original sequence. Notably, GMG_01_SCR shows a peculiar sequence with all the charged residues concentrated on one terminus, demonstrating that the charge ‘spatial’ distribution is an important feature of functional alpha-helical AMPs. As expected, no antimicrobial activity was observed for the GMG_03 peptide, despite its alpha-helical secondary structure. This is likely related to the negative net charge of the peptide at physiological pH, which may not favour its adhesion to the bacterial cell surface.

**Table 4:** Tested peptides in this article.

				Prediction Fitness		MBC	
Name	Sequence	Size	MW	AMP	All-Alpha	P.aeruginosa	S.aureus
GMG_01	VKSWIRKLVHR	11	1421.74	0.80	0.91	1 µM	1 µM
GMG_02	WLKGLIKFIR	10	1273.62	0.72	0.79	2 µM	2 µM
GMG_01_SCR	KRRKWHSVVLI	11	1421.74	0.45	0.55	16 µM	16 µM
GMG_03	EHMDRILAQLL	11	1338.6	0.20	0.87	>50 µM.	>50 µM
CM18^1^	KWKLFKKIGAVLKVLTTG	18	2030.55	0.93	0.53	2 µM	0.5 µM
CM12	WKLFLKAVKKLL	12	1486.93	0.99	0.92	2 µM	0.5 µM
GMG_05Z	HZMRILAQLZKR	12	1527.93	0.93	0.94	0.25 µM	0.125 µM

A series of peptides where synthesized and tested in order to asset AMP activity.

1 peptide from [8].

Z: Norleucine.

### AMP optimization and inclusion of non-natural AA

Optimization of an existing antimicrobial peptide (CM_18_) was performed, in order to obtain an improved system at shorter length, thus with easier synthesis requirements. To this aim, a size constraint was added to preferentially select peptides with sequences shorter than 14 residues. Furthermore, a third objective was added to avoid an excessive difference from the original peptide, the Smith-Waterman normalized score (**Equation 5** in the Materials and Methods). This score measures the similarity between two amino acidic sequences, normalized by the sequence size and requires a measure of the similarity between two AAs. Instead of the commonly used BLOSUM or PAM, a score matrix obtained by the Euclidean distance between each amino-acid z-scale values was used (**Figure S2**). Interestingly, residues with similar physicochemical characteristics grouped together. This facilitates single-AA substitutions, particularly regarding non-natural residues. The sequence of CM_18_ was intentionally removed from the AMP dataset in order to avoid improper influence on the optimization process. A sequence of 12 AAs was selected (CM_12_), as reported in **Table 4**. When tested for its MBC, CM_12_ retained full activity, notwithstanding a nearly 30% decrease in chain length.

**Figure 3:**
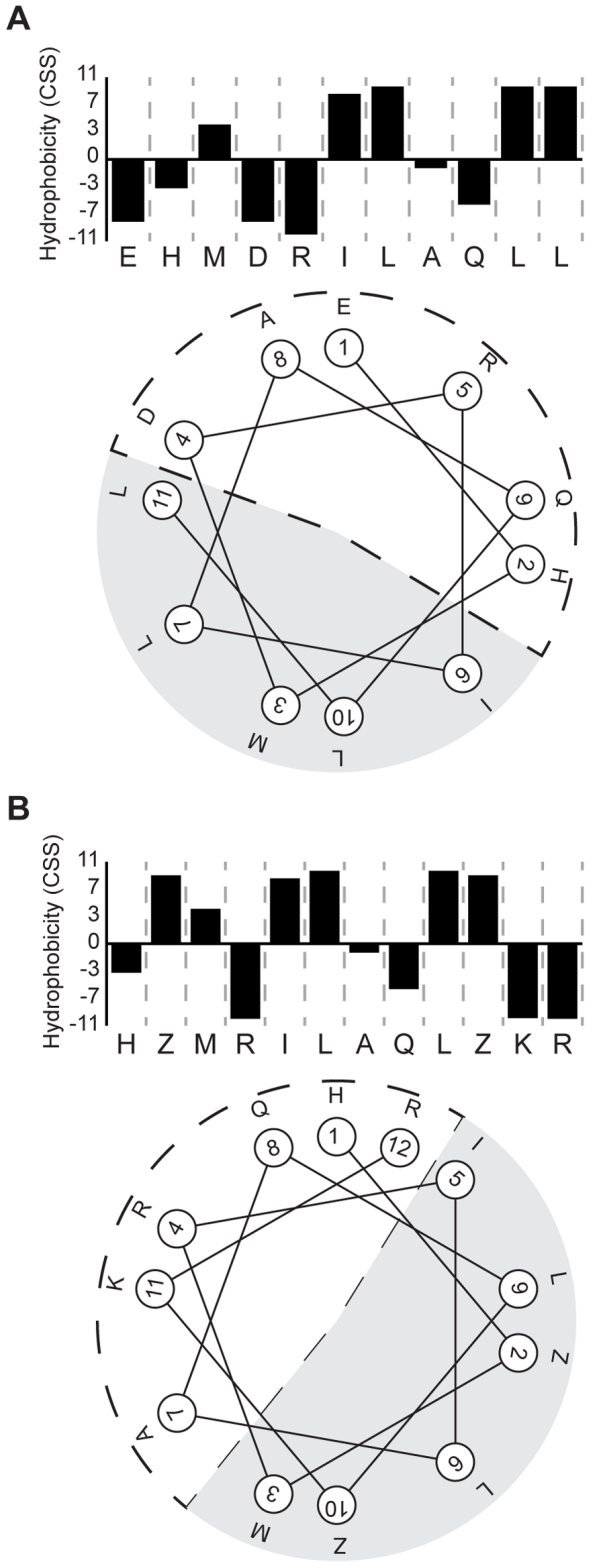
Helical wheel projections of GMG_03 and GMG_05Z. The polar section is indicated by a dotted line, while the hydrophobic face by gray shading. On top of each helical projection a hydrophobicity profile calculated with the CSS scale [53] is schematized. A) GMG_03 peptide. B) GMG_05Z peptide, with non-natural AAs.

Finally, starting from the non-active ab-initio peptide - GMG_03 – the sequence was optimized for antimicrobial activity and all-alpha structure. In the optimization process, the amino-acid alphabet was extended to non-natural elements, including all the 87 AAs listed by the z-scale descriptors. The process was terminated after 300 generations, as described in SI (**Text S1**). From the candidate list, a sequence with two non-natural substitutions was chosen from a list of feasible solutions and synthesized. The selected peptide contains two norleucine (Nle) residues, one substituting the original leucine residue and the other one in the proximity of the C-terminus. MBC assays demonstrate an enhanced antimicrobial activity, significantly higher than the original one. It is worth noting that the overall net charge increased due to the elimination of two negatively charged and the insertion of two positively charged residues. This may facilitate the initial attachment of the peptide to the membrane. The new residue distribution confers a high amphipathicity to the resulting peptide sequence (**Figure 3**). Interestingly, Nle is an artificial AA frequently used in antimicrobial peptide design for research purpose [3] as well as in clinical studies [10]. Analysis of the AA z-scores heatmap (**Figure S2**) revealed a clusterization of Nle with its natural precursor leucine, justifying the substitution choice.

### Molecular dynamics simulations

**Figure 4:**
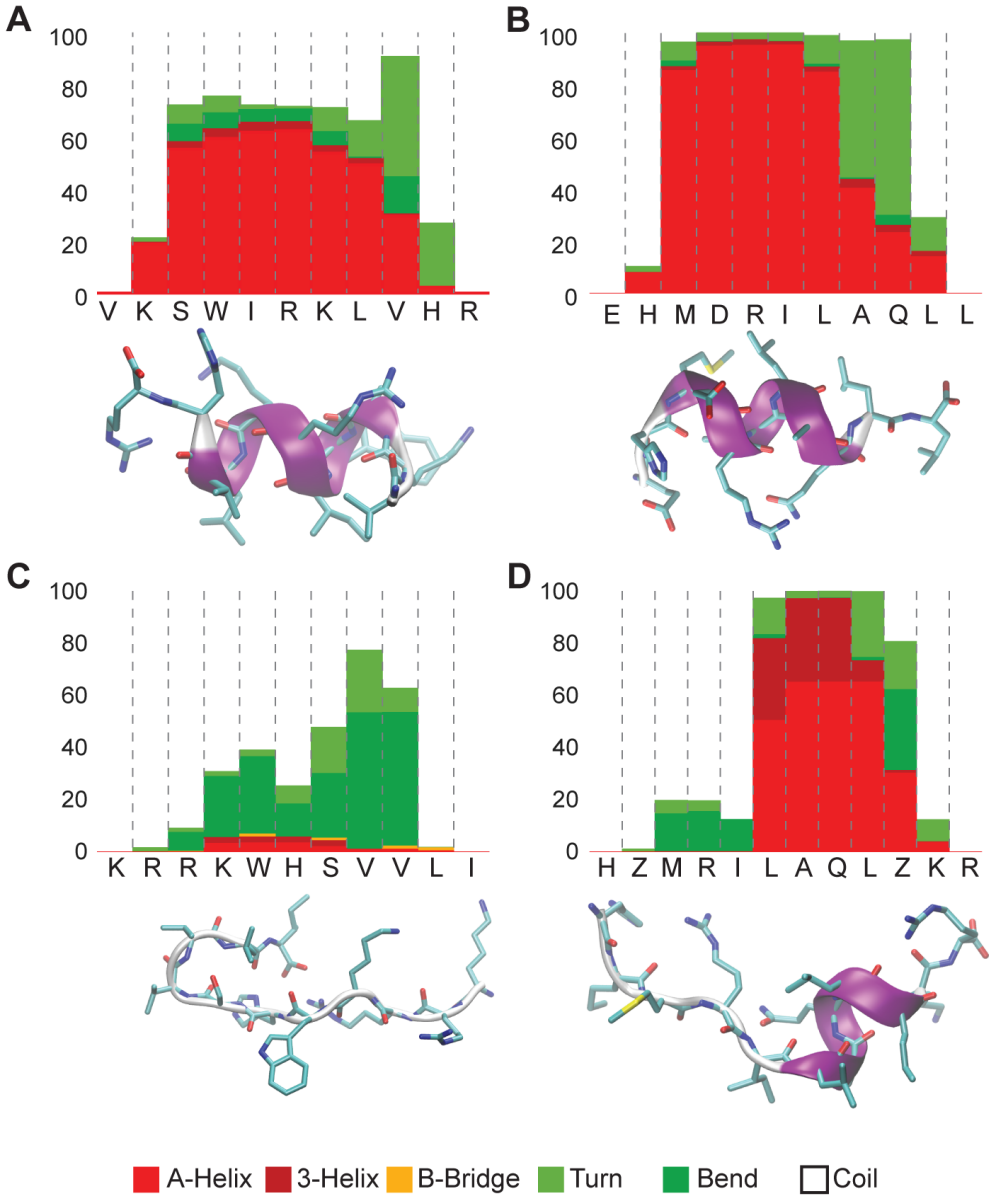
Secondary structure content by MD. In order to assess secondary structure prediction, MD simulations were performed on some peptides. A) GMG_01 in TFE/water. B) GMG_03 in water. C) GMG_01_SCR in TFE/water. D) GMG_05Z in TFE/water.

MD simulations were performed on a selected subset of peptides (GMG_01, GMG_03, GMG_01_SCR and GMG_05Z) to assess the accuracy of the proposed algorithm in terms of structural prediction. Different solvent conditions were simulated, either water or TFE/water mixture. The latter condition is known to stabilize secondary-structure elements and to partially account for the hydrophobic environment inside the lipid bilayer. The percentage of alpha-helix structure vs. other secondary-structure motives was monitored during 700 ns of molecular dynamics after suitable equilibration. The MD simulations fully support the structural predictions of the algorithm (**Figure 4**). In particular, both GMG_01 in TFE/water and GMG_03 in pure water assume rather stable helical conformations. GMG_01_SCR in TFE/water, by contrast, displays negligible alpha-helix propensity again confirming the algorithm prediction. GMG_01 was simulated both in pure water (**Figure S3**) and in TFE/water mixture. Remarkably, the simulations predict a high percentage of helical structure only in the latter condition, as is the general behavior of linear alpha AMPs [3]. Finally, the TFE/water MD simulations of the NLE-containing peptide (GMG_05Z) show the formation of a very stable helical portion, but limited to residues 6 to 9 in the sequence, while the N-terminal portion results completely unstructured. Though MD simulations in the μs range should generally be sufficient for adequate exploration of the conformational landscape in the short peptides examined, it is not possible to rule out slower folding time for some sequences. In particular, in the GMG_05Z case, the simulated time was extended to 2 µs showing an unfolding of the helix followed by folding into an enlarged alpha-helix, also comprising residues 4 and 5 in the sequence. These results are shown in **Figure S5**, also reporting the time series of secondary structure motives for GMG_01, GMG_01_SCR and GMG_03.

### Confocal imaging analysis

**Figure 5:**
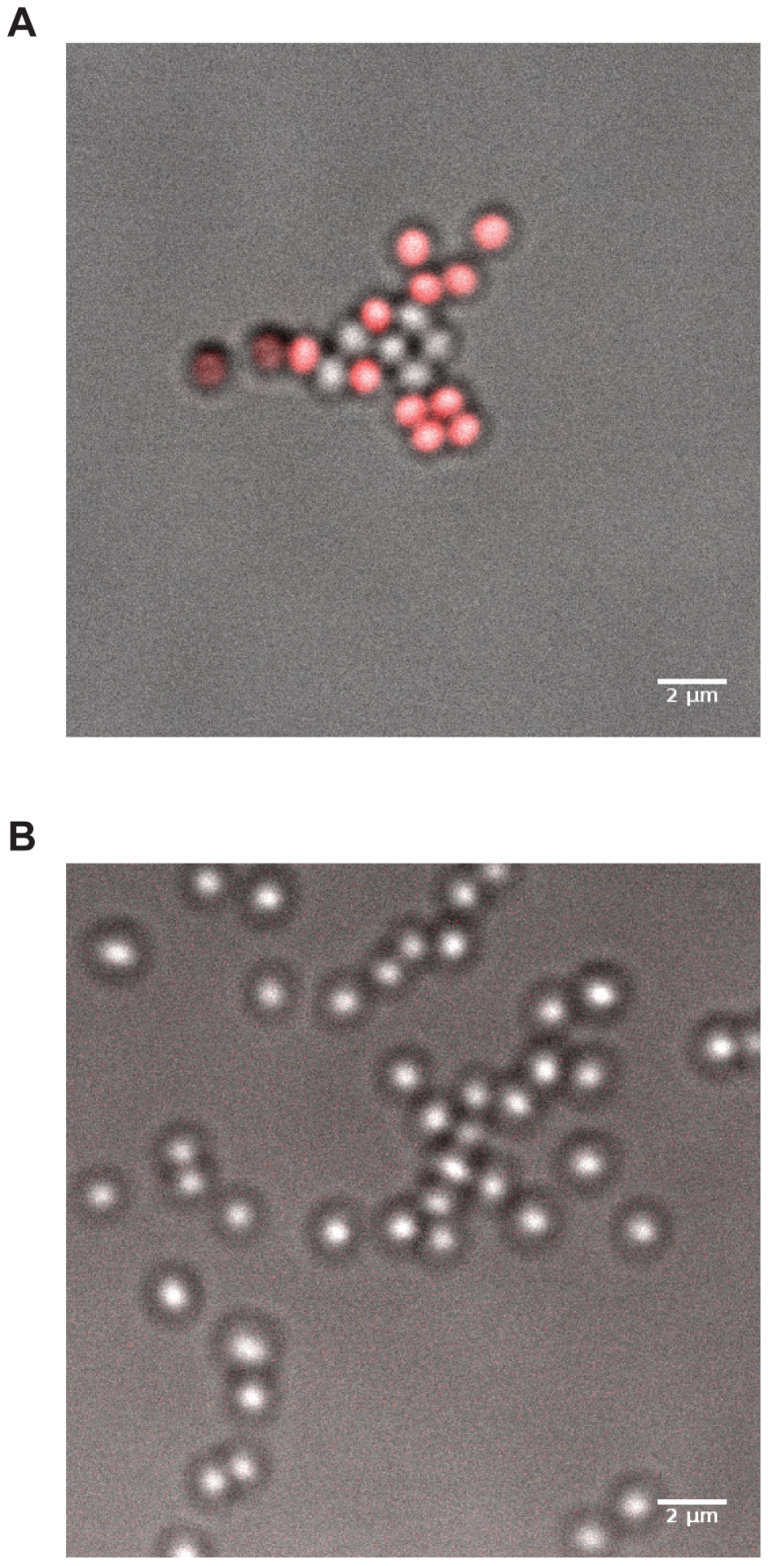
Confocal pictures of bacteria treated with ATTO633-labeled peptides. A) Bacteria treated with GMG_05Z-ATTO633 peptide. B) Bacteria treated with GMG_03-ATTO633 peptide.

In order to analyze the mechanism of action of GMG_05Z compared with the original peptide GMG_03, it was of significant interest to determine their localization in bacteria following treatment. GMG_05Z and GMG_03 analogues with a C-terminal cysteine-atto633 insertion were synthesized and purified. Fluorescence and confocal microscopy were performed after treatment of an ATCC *S.aureus* strain (ATCC33591) with both peptides separately. To determine the influence on the antimicrobial activity of the C-terminus cysteine-atto633 insertion, MBC test were repeated. No significant variations were detected for GMG_03, while GMG_05Z MBC was twofold greater (0.25 µM), indicating that the addition of cysteine-atto633 had a minimal effect on the antimicrobial activity. MBC results are summarized in **Table S4** of the supporting information. As a further control on the structural influence of the N-terminal cysteine residue, GMG_01 and GMG_03 MD simulations were repeated adding this AA to the sequence. The results (**Figure S3**) confirm that the effect of this addition is very limited. Confocal images of *S.aureus* exposed to GMG_05Z revealed its ability to make contact with the membrane (**Figure 5A**). As expected, the inactive peptide (GMG_03) was instead unable to interact with bacteria, as shown in **Figure 5B**. Further studies are required in order to understand whether GMG_05Z acts by forming a transient pore or via metabolic mechanisms.

### Conclusions

In this work, a rapid and intuitive method for virtual screening of antimicrobial candidates was introduced. The method can be successfully applied to *ab-initio* prediction as well as peptide optimization with natural and non-natural AAs. Three different types of topological descriptors were applied for model construction of antimicrobial peptides and all-alpha peptides. The novel mMACC algorithm retained the best performance (as assessed by its highest MCC values), thus lowering the number of needed descriptors. Furthermore, the identification of the optimal complexity of auto and cross covariance descriptors was achieved automatically by IFS, eliminating the tedious process of manual feature selection. The use of physicochemical descriptors allows the analysis and prediction of non-natural AAs insertions, extending the flexibility in peptide design. The *ab-initio* peptide prediction demonstrated the high degree of flexibility of the multi-objective evolutional algorithms (MOEA) approach, in which constraints and objectives can be added depending on the needs. The potential of this approach was demonstrated by transforming a non-antimicrobial peptide into a highly active AMP, using non-natural AAs. Finally, virtual screening was combined with MD simulations to gain insight into the structural properties of the predicted AMPs, and thus provide the molecular basis for understanding peptide-membrane interaction mechanisms. In conclusion, the combination of chemophysical descriptors and MOEA confers an elevated flexibility to antimicrobial peptide design, permitting to select highly active molecules.

## Materials and Methods

### Datasets

Two different datasets, Dataset A and B, were constructed for model training and validation. Dataset A consists of antimicrobial peptides with a sequence length ranging from 11 to 40 residues extracted from YADAMP and CAMP databases [13], [27]. After removal of peptides with disulfide bridges and non-standard residues sequences, 1884 peptides were left. The negative dataset was populated with non-secretory sequences randomly extracted from UniProt database, without ‘antimicrobic’ annotation and with a length ranging from 11 to 40 AAs. Dataset B represents all-alpha helical peptides. The CB513 dataset, a non-redundant set of 513 well-defined proteins [33] was used in a first step for extraction of all-alpha, all-beta and all-coil domains. Then a number of random sequences were extracted from the same database in order to account for mixed secondary structure states. The final dataset was then built using the simplest partition of the space into alpha and non-alpha peptides. For both datasets, a homology cutoff was imposed to exclude similar peptides in order to avoid redundant data that could influence the prediction performance. Peptides showing equal to or greater than 70% sequence identity to any other in the dataset were identified and removed by the CD-HIT program [34]. Final datasets composition is summarized in **Table 5**.

**Table 5:** Final dataset composition.

Dataset	Positive dataset	Negative dataset
Dataset A	892 antimicrobial peptides extracted from YADAMP database.	1800 non-secretory random sequences extracted from UniProt database without ‘antimicrobic’ tag.
Dataset B	972 all-alpha peptide fragments.	2126 peptide fragments in all-coil, all-sheet and mixed conformation.

Positive and negative composition of each training set.

### Data encoding

Global and topological descriptors were utilized in order to encode peptide sequences. Peptide charge at different pH conditions, isoelectric point and the number of positive and negative charges were used to describe charge-related characteristics. The z-scale moment (μZ_i_), an extension of Eisenberg's hydrophobic moment equation [35], is introduced to represent z-scales distribution along peptide sequences.
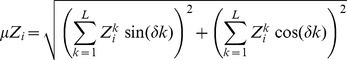

**Equation 2**. Z-scale moment.

In **Equation 2**, *δ* is the angular frequency of the AA residues forming the structure (100° for alpha helix); *k* is the number of the particular residue examined, *L* is the length of the sequence and *Z_i_^k^* is the z_i_-scale value of the *k^th^* AA. In particular, μZ_1_ represents a measure of the hydrophobicity distribution along peptide sequence. Average sum of z-scale descriptors has been successfully used in QSAR analysis of bioactive peptides [36], as it gives a general description of peptides physicochemical main features [37]. The aim of the study was to develop an alignment-independent method, therefore position specific score matrix (PSSM) as well as amino acidic and pseudo-amino acidic sequence descriptors were avoided. Both in the global and topological descriptors, Z-scale values were mean-centered and scaled prior to their use, as described by the following equation:
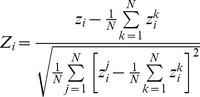

**Equation 3**. Z-scale descriptor normalization.

Where Z_i_ is the i^th^ descriptor of z-scales variables, z_i_ is the original z-scale value (from [21]) and N is the number of AAs in the z-scales descriptors table. The final list of descriptors is summarized in **Table 1**.

### Feature selection and model generation

In this study, the Random Forest algorithm (RF), implemented in the software suite WEKA [38], was adopted as prediction engine. During the evaluation procedure, nine different variables combinations were tested for model building. In particular, the number of trees in the forest (M) and the number of random variables used for each tree (T). Each model performance was measured with a 10-fold cross-validation analysis, where each dataset was divided into 10 parts - 9 parts for model learning (training) and the remaining part for validation (testing). Four performance measures were used: true positive rate for sensitivity, false positive rate for selectivity, predictive accuracy and MCC, as defined below.
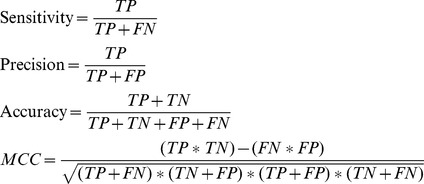

**Equation 4**. Performance evaluation equations

Where *TP*, *TN*, *FP* and *FN* are the number of true positive, true negative, false positive and false negative, respectively, resulting from the model. MCC is an important index used to evaluate the performance of the predictor when the dataset is not balanced [39]. In order to obtain a non-redundant set of descriptors, the Maximum Relevance, Minimum Redundancy (mRMR) method [31] was employed to sort features in descending order of importance. Incremental Feature Selection (IFS) [40] was applied to the sorted descriptors list by incrementing consecutively the number of descriptor by 5. Each descriptor set thus obtained was evaluated by tenfold cross-validation and the IFS curve was plotted to unveil the relation between the performance of the model and the feature subset. The optimal feature subset is defined as that showing the highest MCC value (**Figure 1**); the selected model was used for peptides classification. The hierarchical list of the final descriptors for Dataset A and Dataset B is shown in **Table S2** and **Table S3**, respectively.

### Sequence similarity

For peptide optimization, a supplemental objective representing sequence similarity was added. Sequence similarity is defined by the Smith-Waterman score between the respective peptide sequences [41]. Since the Smith-Waterman score is dependent on input sequences length, the final score was normalized between 0 and 1 by dividing by the maximum score of the two self-alignments, as shown in **Equation 5** [42].
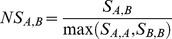

**Equation 5**. Smith-Waterman normalized score

Here, S_A,B_ is the similarity score between sequence A and B, S_A,A_ and S_B,B_ are the self-alignment score of sequence A and sequence B, respectively. In order to consider not only the identity between two amino acidic positions, a score matrix was defined by calculating the Euclidean distance between the five auto-scaled z-scale values of each AA pairs. The score was then normalized between 0 and 1, where 1 is the identity. For visualization purpose, the resulting matrix was analyzed with R [43] and a heat map was produced, calculated as Log_2_ of the inverse AA distance normalized by AA median value (**Figure S2**).

### Peptide synthesis, purification and labeling

All peptides were prepared by solid-phase synthesis using Fmoc chemistry on an automatic peptide synthesizer and the crude peptides were purified by RP-HPLC, as previously described [8]. The cysteine residue added to the C-terminus of some peptides provided a sulfhydryl group for further ligation to the atto-633-maleimide fluorophore. The labelling of purified peptides was performed by incubating for 3 h with a 3-fold molar excess of atto-633-maleimide (ATTO-TEC GmbH, Germany), 150 mM PBS buffer, TCEP, at pH 7.4. Finally, atto-633-labeled peptides were purified by HPLC and then lyophilized overnight. The correct purified product was confirmed by electrospray mass spectroscopy with an API3200QTRAP a Hybrid Triple Quadrupole/Linear Ion Trap (ABSciex, Foster City, California, USA). Peptides were stored at −80 C.

### Bactericidal assay

Antibacterial activity of designed peptides was evaluated by a liquid microdilution assay in 10 mM sodium phosphate buffer (SPB), pH 7.4, as described previously [44]. Briefly, *S. aureus* ATCC33591 and *P. aeruginosa* ATCC27853 were grown in tryptone soy broth (TSB; Oxoid). Exponentially growing bacteria were resuspended in SPB to obtain a density of 1∧10^6^ colony forming units (CFU)/ml and exposed to different concentrations of peptide, ranging from 64 µM to 0.25 µM. After incubation of peptides for 1.5 h at 37°C, 0.2 ml of 10-fold serial dilutions of each samples were plated onto tryptone soy agar. As a control, bacteria were also incubated in the absence of peptides. After 24 h incubation at 37°C the number of CFU was assessed. Bactericidal activity was evaluated as minimal bactericidal concentration (MBC) defined as the lowest peptide concentration at which a reduction in the CFU/ml numbers of > = 3 logs was observed after 1.5 h of incubation in three independent experiments.

### Molecular dynamics simulations

Molecular dynamics simulations of selected peptide sequences (GMG_01, GMG_03, GMG_01_SCR and GMG_05Z) were performed with GROMACS 4.5.5 [45]. The force field used was Amber ff99SB-ILDN-NMR [46]. Random configurations of the peptides were solvated either with a box of TIP3P water molecules, or with a mixture of TFE (trifluoroethanol) and TIP3P water molecules (4 water molecules each TFE molecule, in order to obtain a ∼50% vol/vol solution). TFE force field parameters were taken from ref [47]. RESP HF/6-31G* charges of TFE and NLE (Norleucine) were taken from ref [47] and [48] respectively, while the other force field terms were taken from the already existing parameters. Either Cl^−^ or Na^+^ ions were added to neutralize the system. The truncated octahedron solvation box dimension was chosen in order to keep a distance of at least 8 Å between the peptide and the box faces, and periodic boundary conditions were applied.

For each examined peptide, simulations were performed under constant temperature (300 K) and pressure (1 atm) conditions, using the Nose-Hoover ensemble [49] for temperature coupling (τ = 0.5 ps) and the Parrinello-Rahman ensemble [50] for pressure coupling (τ = 5 ps). The timestep was set at 2 fs, and the bonds involving hydrogen atoms were constrained using LINCS [51]. After an equilibration phase of 300–500 ns, the production runs lasted for 700 ns, and peptide snapshots were recorded each 10 ps. These 700 ns production runs were used for secondary structure analysis, performed using DSSP [52].

### Confocal imaging

As previously described, *S. aureus* ATCC33591 strain was grown in tryptone soy broth and exponentially growing bacteria were resuspended in SPB to obtain a density of 1∧10^8^ CFU/ml and exposed to 2.5 µM concentration of peptide labeled with ATTO633 (**Table S2**). After incubation for 15 min at 37°C, 5 µL of the solution were spotted onto a slice of 1% water agarose gel and placed on a glass bottom petri dish. Images were acquired using a Leica TCS SP5 SMD inverted confocal microscope (Leica Microsystems AG) interfaced with a HeNe laser for excitation at 633 nm and the sample was viewed with a 63×1.2 NA water immersion objective (Leica Microsystems). The pinhole aperture was set to 0.5 Airy. All data collected were analyzed by ImageJ software version 1.44o.

## Supporting Information

Figure S1 **MOEA scheme.** A) Machine learning model construction. The initial dataset is encoded with global and topological descriptors. A sorted list of descriptors is composed and the IFS method is applied to construct the final model. B) Each solution is represented by a chromosome and treated as an individual. Starting from an initial random population, objectives are evaluated for each individual. Afterwards, parents are picked from the population in order to generate new child with crossover and mutation operations. Objectives are calculated for the new child and the new population is selected. The main loop is repeated for a fixed number of generations or until convergence is reached. C) NSGA-II Solution ranking. The parent population Pt and offspring population Qt are combined to form an intermediate population Rt of size 2N. Non-dominated individuals are inserted in the best ranking fronts (dark gray), the remaining ones are sorted by the crowding distance. The new parent population Pt+1 is created by choosing individuals of best ranked fronts first followed by the next-best and so on, till we obtain N individuals.
(TIF)

Figure S2 **Clustered heat map of AAs z scores.** For each AA pairs, the Euclidean distance between the five auto-scaled z scores was calculated. For visualization purpose, the resulting matrix was plotted as a heatmap, calculated as Log_2_ of the inverse AA distance normalized by AA median value.
(TIF)

Figure S3 **Secondary structure content by MD.** In order to asset secondary structure prediction, additional MD simulations were performed on some tested peptides with an additional cysteine residue or on different conditions. A) GMG_01 in water. B) GMG_03 in water with an additional cysteine residue GMG_01_SCR in TFE/water. C) CM12 in water/TFE.
(TIF)

Figure S4 **Feature analysis of Dataset A and B descriptors.** A) Z-scale distribution of AMPs descriptors (dataset A). B) Z-scale distribution of alpha-helix descriptors (dataset B). C) Z-scale descriptor distribution for each lag in dataset A. D) Z-scale descriptor distribution for each lag in dataset B.
(TIF)

Figure S5 **Time series of secondary structure motives from the MD simulations.** A) GMG_01 in TFE/water mixture, B) GMG_01 in water, C) GMG_01_SCR in TFE/water D) GMG_03 in water, E) GMG_05Z in TFE/water.
(TIF)

Table S1 **List of commercially available and clinical trial AMPs.** *: Fox JL (2013) Antimicrobial peptides stage a comeback. Nature biotechnology 31: 379–382.
(DOC)

Table S2 **Hierarchical list of descriptors for Dataset A.** Final list of selected descriptors used for AMP model training.
(DOC)

Table S3 **Hierarchical list of selected descriptors for Dataset B.** Final list of selected descriptors used for all-alpha model training.
(DOC)

Table S4 **Labelled peptides MBC.** In order to analyze the mechanism of action, two peptides with a C-terminus cysteine-ATTO633 insertion were synthesized and purified. MCB tests were repeated to determine the influence on the antimicrobial activity, indicating a minimal effect.
(DOC)

Text S1 **Description of multi-objective evolutional algorithm and descriptor distribution analysis.**
(DOC)
