# Supplementary figures and images for: Antimicrobial Peptides Design by Evolutionary Multiobjective Optimization
Source: PLoS Comput Biol. 2013 Sep 5;9(9):e1003212. doi: 10.1371/journal.pcbi.1003212 (PMC3764005; doi:10.1371/journal.pcbi.1003212)

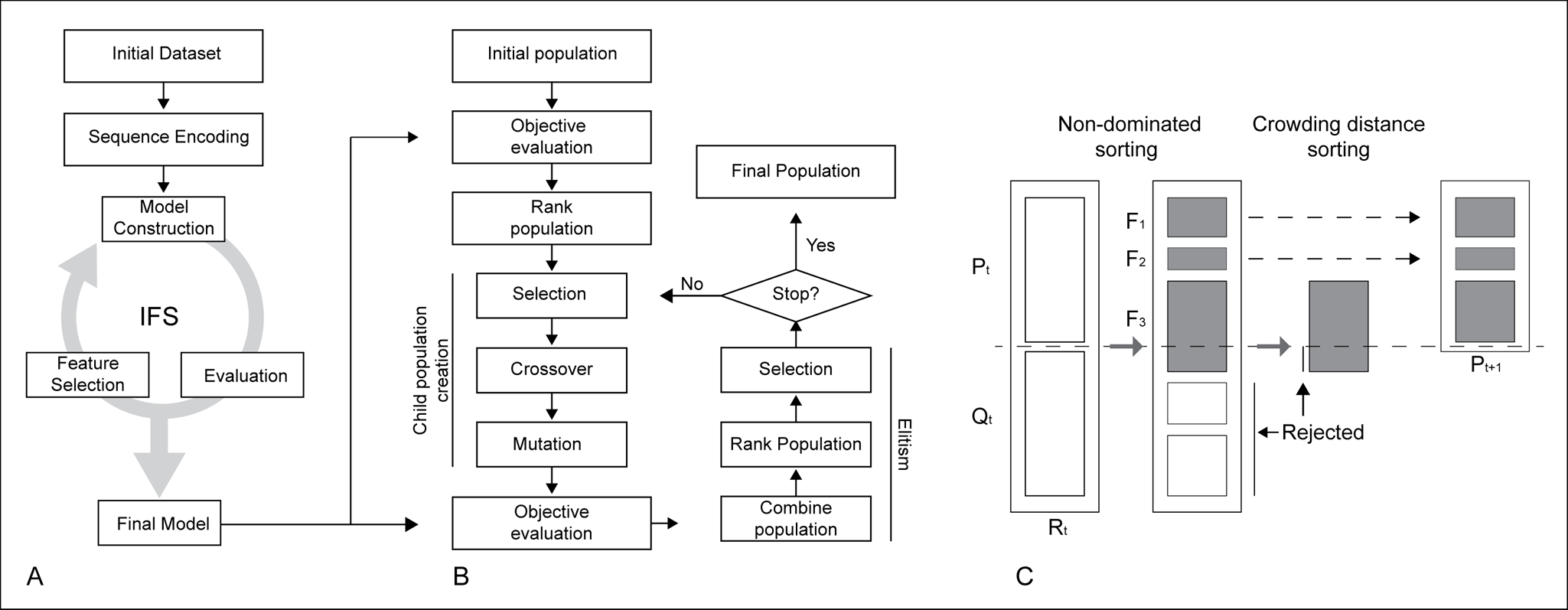

Supplement: Figure S1 — MOEA scheme. A) Machine learning model construction. The initial dataset is encoded with global and topological descriptors. A sorted list of descriptors is composed and the IFS method is applied to construct the final model. B) Each solution is represented by a chromosome and treated as an individual. Starting from an initial random population, objectives are evaluated for each individual. Afterwards, parents are picked from the population in order to generate new child with crossover and mutation operations. Objectives are calculated for the new child and the new population is selected. The main loop is repeated for a fixed number of generations or until convergence is reached. C) NSGA-II Solution ranking. The parent population Pt and offspring population Qt are combined to form an intermediate population Rt of size 2N. Non-dominated individuals are inserted in the best ranking fronts (dark gray), the remaining ones are sorted by the crowding distance. The new parent population Pt+1 is created by choosing individuals of best ranked fronts first followed by the next-best and so on, till we obtain N individuals. (TIF) [file pcbi.1003212.s001.tif]

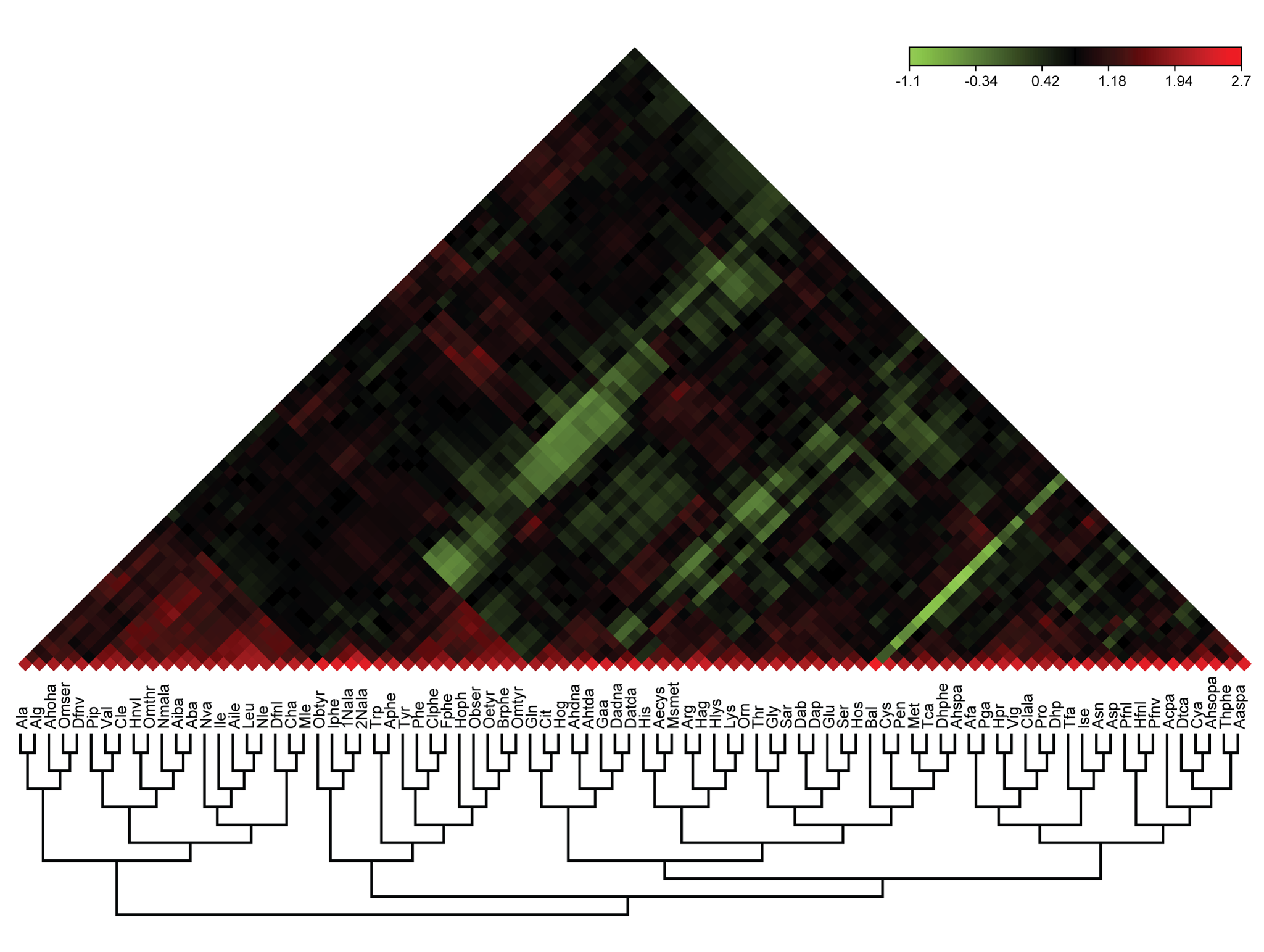

Supplement: Figure S2 — Clustered heat map of AAs z scores. For each AA pairs, the Euclidean distance between the five auto-scaled z scores was calculated. For visualization purpose, the resulting matrix was plotted as a heatmap, calculated as Log2 of the inverse AA distance normalized by AA median value. (TIF) [file pcbi.1003212.s002.tif]

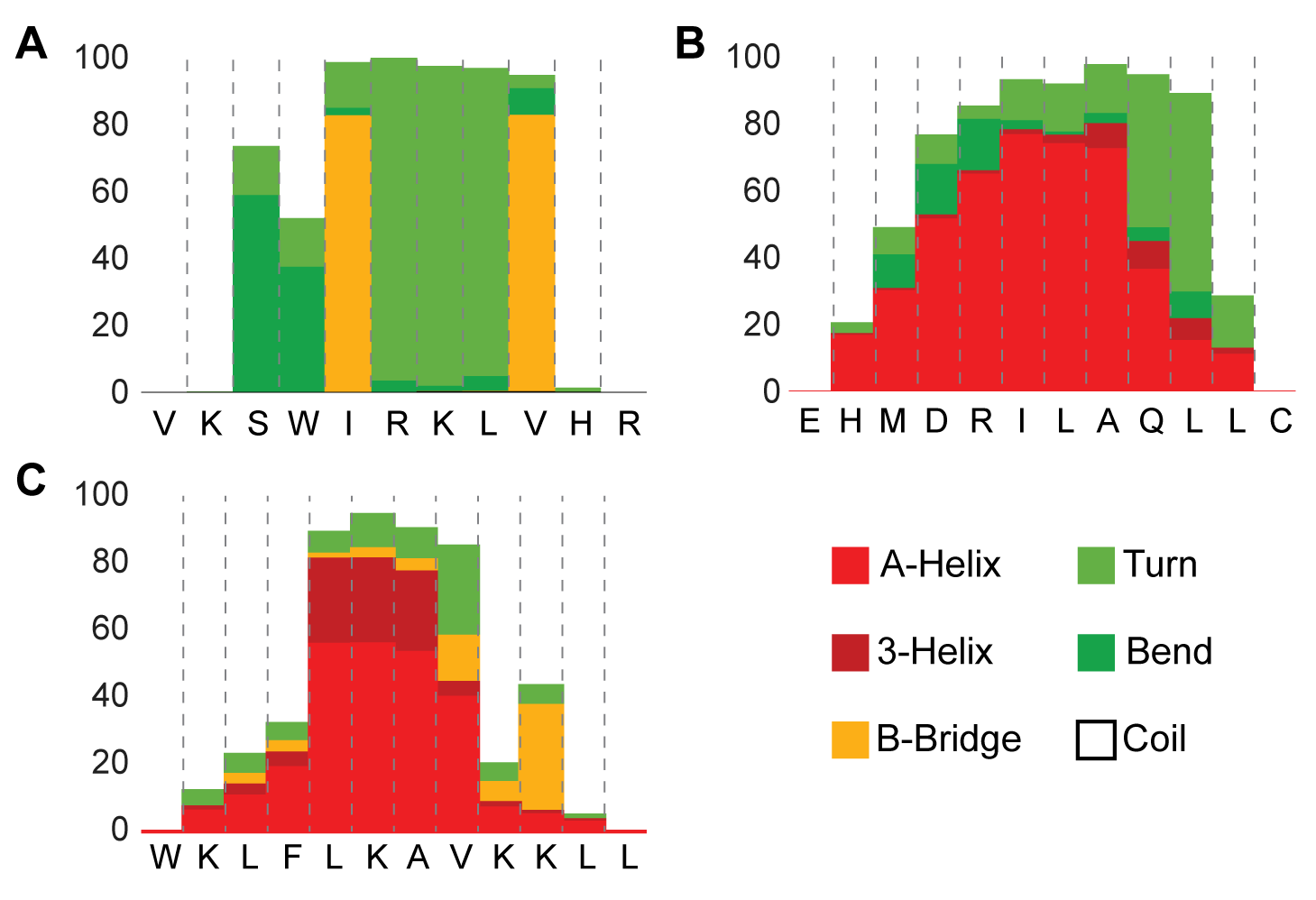

Supplement: Figure S3 — Secondary structure content by MD. In order to asset secondary structure prediction, additional MD simulations were performed on some tested peptides with an additional cysteine residue or on different conditions. A) GMG_01 in water. B) GMG_03 in water with an additional cysteine residue GMG_01_SCR in TFE/water. C) CM12 in water/TFE. (TIF) [file pcbi.1003212.s003.tif]

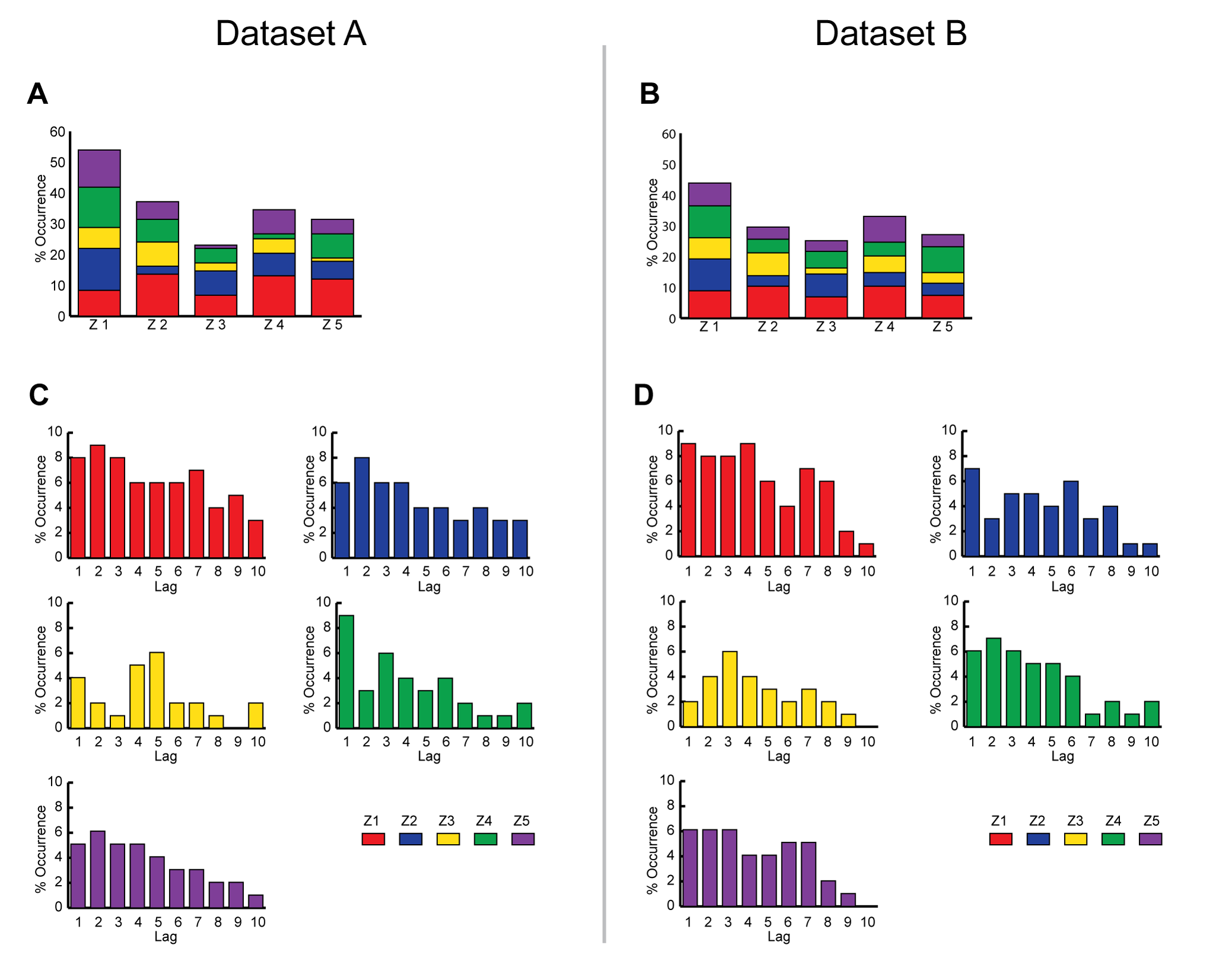

Supplement: Figure S4 — Feature analysis of Dataset A and B descriptors. A) Z-scale distribution of AMPs descriptors (dataset A). B) Z-scale distribution of alpha-helix descriptors (dataset B). C) Z-scale descriptor distribution for each lag in dataset A. D) Z-scale descriptor distribution for each lag in dataset B. (TIF) [file pcbi.1003212.s004.tif]

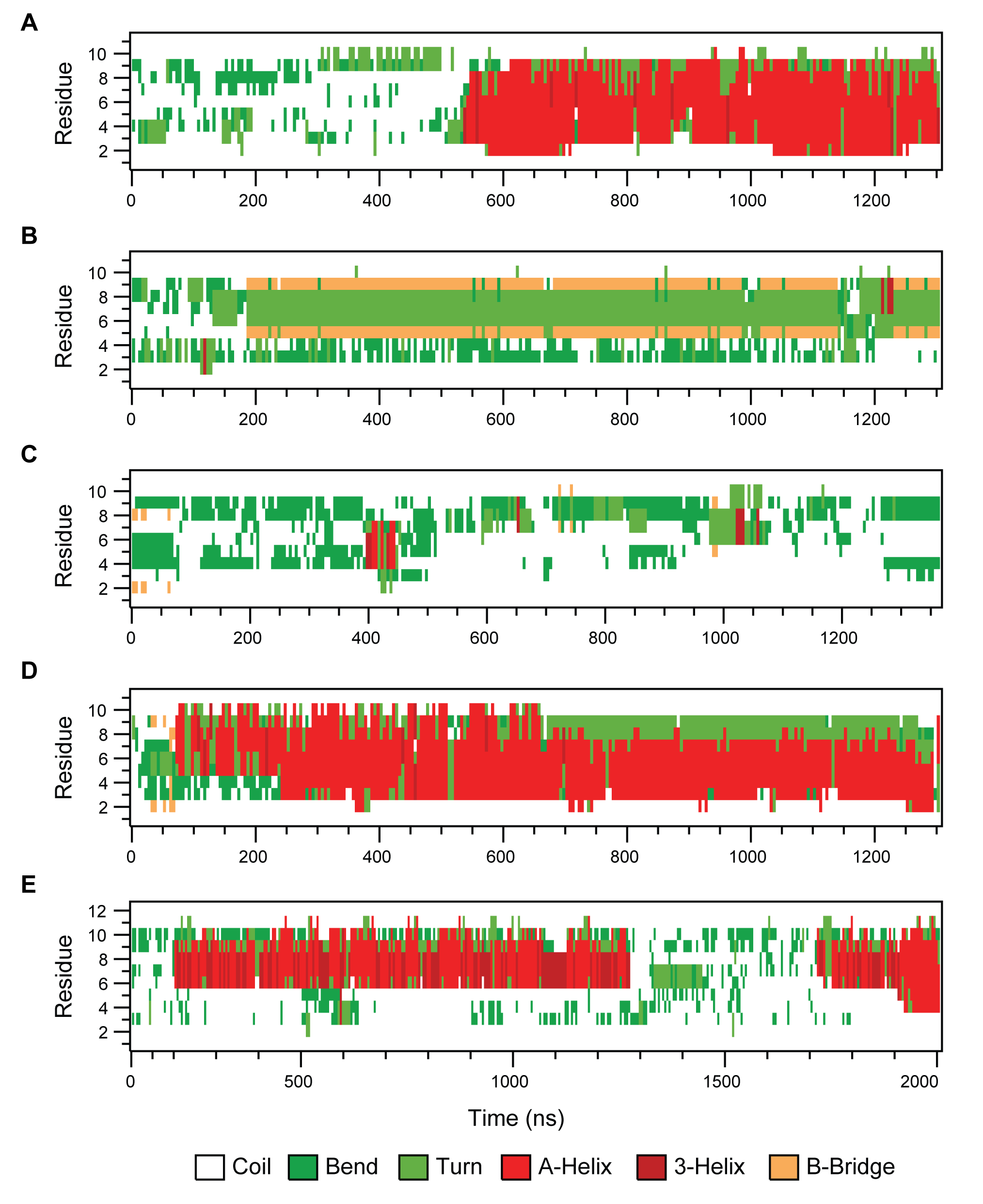

Supplement: Figure S5 — Time series of secondary structure motives from the MD simulations. A) GMG_01 in TFE/water mixture, B) GMG_01 in water, C) GMG_01_SCR in TFE/water D) GMG_03 in water, E) GMG_05Z in TFE/water. (TIF) [file pcbi.1003212.s005.tif]
