# Supplementary material for: Antimicrobial Peptides Design by Evolutionary Multiobjective Optimization
Source: PLoS Comput Biol. 2013 Sep 5;9(9):e1003212. doi: 10.1371/journal.pcbi.1003212 (PMC3764005; doi:10.1371/journal.pcbi.1003212)
Supplement: Table S1 — List of commercially available and clinical trial AMPs. *: Fox JL (2013) Antimicrobial peptides stage a comeback. Nature biotechnology 31: 379–382. (DOC) [file pcbi.1003212.s006.doc]

| **Name** | **Company** | **Medical use** | **Stage** |
| --- | --- | --- | --- |
| Daptomycin | Cubist Pharmaceutical | Multi-resistant bacteria skin infection | Commercially available |
| Bacitracin | X-GEN Pharmaceutical (Baciim) | Staphylococcal skin infections | Commercially available |
| Polymyxin B | Emessa LAB (NEOCIN) | Wound treatment and skin infections | Commercially available |
| Magainin peptide/ pexiganan acetate | Dipexium Pharma/MacroChem/Genaera | Diabetic foot ulcers | Phase 3* |
| Omiganan | BioWest Therapeutics/Maruho | Rosacea | Phase 2* |
| OP-145 | OctoPlus | Chronic bacterial middle-ear infection | Phase 2* |
| Novexatin | NovaBiotics | Fungal infections of the toenail | Phase 1* |
| Arenicin | Adenium Biotech | Multi-resistant Gram-positive bacteria | Preclinical* |
