# Supplementary material for: Antimicrobial Peptides Design by Evolutionary Multiobjective Optimization
Source: PLoS Comput Biol. 2013 Sep 5;9(9):e1003212. doi: 10.1371/journal.pcbi.1003212 (PMC3764005; doi:10.1371/journal.pcbi.1003212)
Supplement: Table S3 — Hierarchical list of selected descriptors for Dataset B. Final list of selected descriptors used for all-alpha model training. (DOC) [file pcbi.1003212.s008.doc]

| **#** | **Descriptor** | **#** | **Descriptor** | **#** | **Descriptor** | **#** | **Descriptor** |
| --- | --- | --- | --- | --- | --- | --- | --- |
| 1 | Z2:1_CC_LAG_3 (Max) | 55 | Z2:1_CC_LAG_9 (Min) | 109 | Z1:2_CC_LAG_0 (Max) | 163 | Z1:5_CC_LAG_6 (Min) |
| 2 | Z1_AC_LAG_3 (Min) | 56 | Z1:3_CC_LAG_3 (Min) | 110 | Z5_AC_LAG_5 (Max) | 164 | Z5:2_CC_LAG_5 (Min) |
| 3 | Z4 Moment | 57 | Z1_AC_LAG_6 (Min) | 111 | Sum Of Charges | 165 | Z1_AC_LAG_7 (Max) |
| 4 | Net Charge @ pH 7.0 | 58 | Z5:3_CC_LAG_1 (Max) | 112 | Z2_AC_LAG_5 (Min) | 166 | Z5_AC_LAG_6 (Max) |
| 5 | Z5 Moment | 59 | Z1_AC_LAG_5 (Max) | 113 | Z1_AC_LAG_1 (Max) | 167 | Z4:1_CC_LAG_4 (Min) |
| 6 | Z2 Moment | 60 | Z2_AC_LAG_4 (Min) | 114 | Z4:5_CC_LAG_0 (Max) | 168 | Z2:4_CC_LAG_4 (Min) |
| 7 | Z2:5_CC_LAG_3 (Min) | 61 | Z3_AC_LAG_7 (Min) | 115 | Z4:1_CC_LAG_2 (Min) | 169 | Z2:1_CC_LAG_0 (Max) |
| 8 | Z5_AC_LAG_3 (Max) | 62 | Z1:4_CC_LAG_1 (Min) | 116 | Z1:3_CC_LAG_1 (Min) | 170 | Z3_AC_LAG_6 (Min) |
| 9 | Z3 Moment | 63 | Z5:4_CC_LAG_4 (Max) | 117 | Z1:2_CC_LAG_3 (Min) | 171 | Z1:4_CC_LAG_0 (Max) |
| 10 | Z4:3_CC_LAG_3 (Min) | 64 | Z1:5_CC_LAG_4 (Max) | 118 | Z5:1_CC_LAG_1 (Max) | 172 | Z4_AC_LAG_5 (Min) |
| 11 | Z5:1_CC_LAG_0 (Min) | 65 | Z1:2_CC_LAG_3 (Max) | 119 | Z2:5_CC_LAG_8 (Min) | 173 | Z2_AC_LAG_2 (Min) |
| 12 | Z4:3_CC_LAG_0 (Max) | 66 | Z5:2_CC_LAG_6 (Min) | 120 | Z2:5_CC_LAG_2 (Max) | 174 | Z3:5_CC_LAG_2 (Max) |
| 13 | Z4:2_CC_LAG_7 (Min) | 67 | Z2:1_CC_LAG_2 (Min) | 121 | Z4:3_CC_LAG_4 (Max) | 175 | Z3:1_CC_LAG_7 (Min) |
| 14 | Z1_AC_LAG_1 (Min) | 68 | Z5_AC_LAG_2 (Max) | 122 | Z3:1_CC_LAG_6 (Min) | 176 | Z5:2_CC_LAG_1 (Max) |
| 15 | Z1_AC_LAG_6 (Max) | 69 | Z1_AC_LAG_2 (Max) | 123 | Z4_AC_LAG_6 (Min) | 177 | Z1:4_CC_LAG_5 (Min) |
| 16 | Z5:4_CC_LAG_1 (Max) | 70 | Z4:1_CC_LAG_1 (Min) | 124 | Z3:4_CC_LAG_5 (Max) | 178 | Z2:3_CC_LAG_6 (Min) |
| 17 | Z4:5_CC_LAG_0 (Min) | 71 | Z3:2_CC_LAG_1 (Min) | 125 | Z1_AC_LAG_7 (Min) | 179 | Z3:2_CC_LAG_2 (Min) |
| 18 | Z1:4_CC_LAG_6 (Min) | 72 | Z4:5_CC_LAG_4 (Max) | 126 | Z1_AC_LAG_3 (Max) | 180 | Z5:1_CC_LAG_4 (Min) |
| 19 | Z1:5_CC_LAG_0 (Max) | 73 | Z1:5_CC_LAG_1 (Min) | 127 | Z2_AC_LAG_0 (Min) | 181 | Z5_AC_LAG_4 (Max) |
| 20 | Z3:2_CC_LAG_3 (Min) | 74 | Z3:1_CC_LAG_3 (Max) | 128 | Z5:1_CC_LAG_1 (Min) | 182 | Z1:2_CC_LAG_6 (Min) |
| 21 | Z4:1_CC_LAG_8 (Min) | 75 | Z5:3_CC_LAG_2 (Min) | 129 | Z2:3_CC_LAG_0 (Min) | 183 | Z1:3_CC_LAG_7 (Max) |
| 22 | Z1 Moment | 76 | Z2:1_CC_LAG_0 (Min) | 130 | Z5:3_CC_LAG_6 (Max) | 184 | Z4 mean value |
| 23 | Z1_AC_LAG_0 (Min) | 77 | Z5:4_CC_LAG_0 (Max) | 131 | Z5:2_CC_LAG_8 (Min) | 185 | Z2:1_CC_LAG_5 (Max) |
| 24 | Z5:1_CC_LAG_0 (Max) | 78 | Z5:1_CC_LAG_2 (Max) | 132 | Z1:2_CC_LAG_0 (Min) | 186 | Z2:1_CC_LAG_5 (Min) |
| 25 | Z1:3_CC_LAG_0 (Min) | 79 | Z1:4_CC_LAG_4 (Min) | 133 | Z2:4_CC_LAG_0 (Max) | 187 | Z4:3_CC_LAG_1 (Min) |
| 26 | Z2_AC_LAG_3 (Min) | 80 | Z2:5_CC_LAG_7 (Min) | 134 | Z5:4_CC_LAG_2 (Min) | 188 | Z1:5_CC_LAG_7 (Min) |
| 27 | Z1:4_CC_LAG_0 (Min) | 81 | Z1_AC_LAG_4 (Min) | 135 | Z1:5_CC_LAG_6 (Max) | 189 | Z2:3_CC_LAG_0 (Max) |
| 28 | Z1:5_CC_LAG_5 (Max) | 82 | Z3:2_CC_LAG_4 (Min) | 136 | Z4:1_CC_LAG_5 (Min) | 190 | Z4:2_CC_LAG_9 (Min) |
| 29 | Z4_AC_LAG_1 (Max) | 83 | Z4_AC_LAG_4 (Min) | 137 | Z1_AC_LAG_4 (Max) | 191 | Z1 mean value |
| 30 | Z4:1_CC_LAG_3 (Min) | 84 | Z4:1_CC_LAG_0 (Max) | 138 | Z5:4_CC_LAG_3 (Max) | 192 | Z4:5_CC_LAG_2 (Max) |
| 31 | Z1_AC_LAG_0 (Max) | 85 | Z5 mean value | 139 | Z1:4_CC_LAG_3 (Min) | 193 | Z2:5_CC_LAG_5 (Min) |
| 32 | Z2:4_CC_LAG_5 (Min) | 86 | Z1_AC_LAG_8 (Min) | 140 | Z3:4_CC_LAG_3 (Min) | 194 | Z3:5_CC_LAG_2 (Min) |
| 33 | Z3:4_CC_LAG_0 (Max) | 87 | Z2_AC_LAG_1 (Min) | 141 | Z5_AC_LAG_1 (Max) | 195 | Z4_AC_LAG_3 (Min) |
| 34 | Z1:5_CC_LAG_2 (Min) | 88 | Z4:3_CC_LAG_2 (Max) | 142 | Z2:1_CC_LAG_4 (Min) | 196 | Z5_AC_LAG_7 (Max) |
| 35 | Z5_AC_LAG_0 (Max) | 89 | Z3:1_CC_LAG_2 (Min) | 143 | Z1:3_CC_LAG_4 (Min) | 197 | Z1:2_CC_LAG_6 (Max) |
| 36 | Z3:2_CC_LAG_5 (Min) | 90 | Z5:1_CC_LAG_4 (Max) | 144 | Z4:3_CC_LAG_1 (Max) | 198 | Z1:3_CC_LAG_2 (Min) |
| 37 | Z2:3_CC_LAG_7 (Min) | 91 | Z4_AC_LAG_9 (Min) | 145 | Z2:5_CC_LAG_6 (Min) | 199 | Z3:5_CC_LAG_1 (Max) |
| 38 | Z1_AC_LAG_2 (Min) | 92 | Z5:1_CC_LAG_3 (Min) | 146 | Z1:5_CC_LAG_3 (Min) | 200 | Z2_AC_LAG_7 (Min) |
| 39 | Z4:5_CC_LAG_1 (Max) | 93 | Z2:3_CC_LAG_1 (Min) | 147 | Z4:2_CC_LAG_2 (Min) | 201 | Z1:4_CC_LAG_2 (Max) |
| 40 | Z5:4_CC_LAG_0 (Min) | 94 | Z1:5_CC_LAG_1 (Max) | 148 | Z4:5_CC_LAG_3 (Max) | 202 | Z5:1_CC_LAG_5 (Min) |
| 41 | Z1:5_CC_LAG_3 (Max) | 95 | Z1:2_CC_LAG_1 (Min) | 149 | Z3:2_CC_LAG_2 (Max) | 203 | Z4:2_CC_LAG_3 (Min) |
| 42 | Z1:4_CC_LAG_8 (Min) | 96 | Z4:1_CC_LAG_1 (Max) | 150 | Average of Charges | 204 | Z3_AC_LAG_8 (Min) |
| 43 | Z3:1_CC_LAG_0 (Min) | 97 | Z4:5_CC_LAG_2 (Min) | 151 | Z5:1_CC_LAG_2 (Min) | 205 | Z1:5_CC_LAG_4 (Min) |
| 44 | Z4:1_CC_LAG_0 (Min) | 98 | Z5:2_CC_LAG_0 (Max) | 152 | Z3:2_CC_LAG_6 (Min) | 206 | Z5:4_CC_LAG_6 (Min) |
| 45 | Z4:2_CC_LAG_4 (Min) | 99 | Z2:1_CC_LAG_3 (Min) | 153 | Z5:1_CC_LAG_7 (Max) | 207 | Z3:1_CC_LAG_4 (Min) |
| 46 | Z1:2_CC_LAG_1 (Max) | 100 | Z5:1_CC_LAG_3 (Max) | 154 | Z2:1_CC_LAG_7 (Min) | 208 | Z2:5_CC_LAG_3 (Max) |
| 47 | Z2:1_CC_LAG_1 (Min) | 101 | Z1:4_CC_LAG_7 (Min) | 155 | Z3:4_CC_LAG_4 (Max) | 209 | Z5:1_CC_LAG_6 (Max) |
| 48 | Z1:5_CC_LAG_2 (Max) | 102 | Z4:2_CC_LAG_5 (Min) | 156 | Z2:3_CC_LAG_2 (Min) | 210 | Z1_AC_LAG_9 (Min) |
| 49 | Z1:5_CC_LAG_0 (Min) | 103 | Z1:5_CC_LAG_7 (Max) | 157 | Z4_AC_LAG_2 (Min) | 211 | Z1:3_CC_LAG_3 (Max) |
| 50 | Z5:1_CC_LAG_5 (Max) | 104 | Z5:4_CC_LAG_2 (Max) | 158 | Z2:1_CC_LAG_2 (Max) | 212 | Z2:3_CC_LAG_5 (Min) |
| 51 | Z3:4_CC_LAG_1 (Max) | 105 | Z1:4_CC_LAG_2 (Min) | 159 | Z1_AC_LAG_5 (Min) | 213 | Z4_AC_LAG_0 (Min) |
| 52 | Z4_AC_LAG_1 (Min) | 106 | Z2:3_CC_LAG_4 (Min) | 160 | Z3:5_CC_LAG_3 (Max) | 214 | Z4:5_CC_LAG_5 (Max) |
| 53 | Z1:2_CC_LAG_2 (Min) | 107 | Z4:1_CC_LAG_7 (Min) | 161 | Z3:1_CC_LAG_1 (Min) | 215 | Z2_AC_LAG_6 (Min) |
| 54 | Z2:5_CC_LAG_0 (Max) | 108 | Z3_AC_LAG_2 (Max) | 162 | Z5:4_CC_LAG_5 (Max) |  |  |
