# Supplementary material for: Antimicrobial Peptides Design by Evolutionary Multiobjective Optimization
Source: PLoS Comput Biol. 2013 Sep 5;9(9):e1003212. doi: 10.1371/journal.pcbi.1003212 (PMC3764005; doi:10.1371/journal.pcbi.1003212)
Supplement: Table S4 — Labelled peptides MBC. In order to analyze the mechanism of action, two peptides with a C-terminus cysteine-ATTO633 insertion were synthesized and purified. MCB tests were repeated to determine the influence on the antimicrobial activity, indicating a minimal effect. (DOC) [file pcbi.1003212.s009.doc]

| Name | Sequence | Size | MW | MBC (*S.aureus*) |
| --- | --- | --- | --- | --- |
| GMG_03_ATTO633 | EHMDRILAQLLC | 12 | 2114.72 | >50 µM |
| GMG_05Z_ATTO633 | HZMRILAQLZKRC | 13 | 2322.95 | 0.25 µM |
