# Supplementary material for: Antimicrobial Peptides Design by Evolutionary Multiobjective Optimization
Source: PLoS Comput Biol. 2013 Sep 5;9(9):e1003212. doi: 10.1371/journal.pcbi.1003212 (PMC3764005; doi:10.1371/journal.pcbi.1003212)
Supplement: Text S1 — Description of multi-objective evolutional algorithm and descriptor distribution analysis. (DOC) [file pcbi.1003212.s010.doc]

SUPPORTING INFORMATIONS

**Multi-objective evolutional algorithm**

Each trained model was used as objective function: a prediction fitness value F*_l_* is generated for each candidate solution *l*, which reflects the confidence assigned to the prediction. The higher the F_l_ is, the more likely that the solution l actually represents a target-class case. Starting from a random parent population *P_t_* of size N with *t*=0, *t* being the number of generation, objectives are calculated for each solution in the population. Each generation is ranked according to the non-domination criterion (**Figure S1**) and N solutions are selected in order to create the new population *P_t+1_*. In order to ensure elitism, the algorithm combines better ranking solutions with dominated solutions up to the final population size. The main loop is repeated for a fixed number of generations or until the algorithm reaches convergence. Starting from an initial population of 500 randomly generated sequence candidates with a length ranging from 10 to 35 amino acids, the NSGA-II algorithm [26] was applied in order to obtain a number of feasible solutions. Crossover probability was set to 0.9 for the first recombination event, whereas the likelihood of subsequent recombination events was set to 0.1. Mutation, insertion and deletion events occurred with a probability of 0.03, 0.01 and 0.01 respectively. The process was terminated after 300 generations, since after this point no significant improvement in the convergence was observed.

**Selected descriptors and their distribution**

The final lists of the selected descriptors used by the two models are reported in **Table S3** (Dataset A) and **Table S4** (Dataset B). Figure S4 reports an analysis of the descriptors in terms of percentage occurrence of z-scales (panels A and B) and of a specific lag value (panels C and D). For instance, Z1 appears in 52% of the descriptors of dataset A (including both AC and CC) as shown in the histogram of panel A. Stacked bars indicate the occurrence of each descriptor coupled with Z1 (Z1:Z1 ~9%,Z1-Z2 ~8%, etc). Furthermore Z1 occurs 8% of times with a lag of 0; 9% with a lag 1, etc (panel C red histograms).

As a general trend, in both datasets, Z1 is the preferred descriptor followed by Z4, whereas Z3 is the least occurring one. Regarding the distribution as a function of the lag, in dataset A local interactions are generally preferred. Interestingly, dataset B displays a rather enhanced occurrence of lag 3 and 4, which nicely correlates with the importance of i, i+3 and i, i+4 interactions in alpha helices. In particular Z3 lag histogram shows a partial periodicity of 4, again in line with the alpha-helix periodic interactions.

Finally, it is worth noting how the descriptors of minimal interactions prevail over those of maximum interactions (104 vs 87 in Dataset A, and 122 vs 82 in Dataset B), supporting the inclusion of the minimum values in the new mMACC (Equation 1 in the main text).
